# Development of High Fat Diet-Induced Hyperinsulinemia in Mice Is Enhanced by Co-treatment With a TLR7 Agonist

**DOI:** 10.3389/fphys.2022.930353

**Published:** 2022-07-06

**Authors:** Rahul M. Kakalij, Del L. Dsouza, Erika I. Boesen

**Affiliations:** Department of Cellular and Integrative Physiology, University of Nebraska Medical Center, Omaha, NE, United States

**Keywords:** metabolic syndrome, lupus, imiquimod, hyperglycemia, insulin, cholesterol

## Abstract

Metabolic syndrome (MetS) is common in Systemic Lupus Erythematosus (SLE) patients and is associated with increased cardio-renal risk. Toll-like receptor 7 (TLR7) stimulation promotes the development of SLE through mechanisms including activating type I Interferon (IFN) and autoreactive B cells. The current study tested whether combined TLR7 agonist treatment and exposure to a high fat, high sucrose “Western diet” intervention affects the early-stage development of SLE or MetS features. Female C57BL/6 mice were untreated or treated with the TLR7 agonist imiquimod (IMQ) and fed a high-fat diet (HFD; fat 42% kcal, sucrose 34% kcal) or control diet (fat 12.6% kcal, sucrose 34% kcal) for 6 weeks. Supporting early-stage induction of autoimmunity, spleen weights were significantly increased and anti-nuclear antibody (ANA) positivity was detected in IMQ-treated mice. Increased body weight, gonadal fat pad mass, and plasma leptin levels were observed between HFD and control animals for both IMQ and untreated mice. However, the increase in these parameters with HFD was slightly but significantly diminished in IMQ-treated mice. Both the HFD and IMQ treatments significantly increased fasting blood glucose levels. Notably, IMQ treatment affected fasting insulin concentrations in a diet-dependent manner, with hyperinsulinemia observed in IMQ-HFD treated mice. Together, this indicates that the IMQ model of SLE is associated with metabolic alterations, impaired glycemic control, and hyperinsulinemia under HFD conditions. This model may be helpful in further investigating the relationship between MetS and SLE, and supports a role of TLR7 signaling in promoting or accelerating the development of dysglycemia and hyperinsulinemia.

## Introduction

Systemic Lupus Erythematosus (SLE) is a complex autoimmune disease with multiple organ involvement. SLE is commonly diagnosed in women during their reproductive years (20–40 years), and, at least in the United States, the disease has a female-male ratio of 9:1 (Margery-Muir et al., 2017). The incidence of SLE varies between geographic locations and by racial or ethnic demographics, with people of black ethnicity having a much higher rate of incidence and prevalence than people of white ethnicity (Rees et al., 2017). Primary features of SLE include autoantibody generation against nuclear material, immune complex deposition in target tissues, including the joints, skin, and kidneys, leading to inflammation and organ damage (Moulton et al., 2017). Impairment in immune complex clearance also occurs and is a crucial pathological mechanism in SLE. Lupus nephritis constitutes one of the most concerning manifestations of SLE, occurring in approximately 30–60% of adult SLE patients and 50–80% of juvenile-onset patients (Pons-Estel et al., 2011; Massias et al., 2020). Up to 20% of lupus patients develop end-stage kidney disease within 10 years of diagnosis (Tektonidou et al., 2016), and a 2016 meta-analysis reported that SLE patients have over a 4-fold increased risk of mortality due to renal disease (Lee et al., 2016). Cardiovascular disease, including coronary artery and cerebrovascular disease, is also a major cause of morbidity and mortality among SLE patients (Burkard et al., 2018; Kostopoulou et al., 2020). Indeed, SLE patients carry at least a 2-fold increased risk of mortality due to cardiovascular disease (Lee et al., 2016), with estimates as high as a 50-fold increased risk of myocardial infarction compared to age- and sex-matched controls (Manzi et al., 1997). The risk of myocardial infarction and cardiovascular mortality have been shown to be even higher in lupus nephritis patients than in SLE patients without lupus nephritis (Hermansen et al., 2017). Part of this elevated risk may be attributable to the observation that SLE patients display premature onset and accelerated progression of atherosclerosis, although the underlying mechanisms are not fully understood (Kostopoulou et al., 2020). Nonetheless, several studies have reported that SLE patients display an elevated risk of cardiovascular events and mortality compared to people without SLE who have similar patterns of traditional cardiovascular risk factors (Goldberg et al., 2009; Ajeganova et al., 2021). SLE patients are often treated with corticosteroids due to their anti-inflammatory properties, but long-term use of these therapies carries a risk of adverse effects on metabolism. As the cardiovascular morbidity and mortality in SLE patients is not fully explained by traditional risk factors alone, this may indicate a synergistic effect of traditional risk factors and SLE, or treatments such as corticosteroids, to promote excess risk (Kostopoulou et al., 2020; Jha et al., 2022). As such, better understanding of cardiovascular disease in the context of SLE is vital to improving patient outcomes.

One of the major drivers of cardiovascular and renal risk in the general population is the “Metabolic Syndrome” (MetS). MetS describes a cluster of metabolic risk factors, including central obesity, insulin resistance, glucose intolerance, hypertriglyceridemia, and hypertension, which promotes the development of type 2 diabetes and cardiovascular disease (Bagheri et al., 2021). Recent meta-analyses report that MetS is approximately twice as common in patients with SLE than in healthy controls (Sun et al., 2017; Hallajzadeh et al., 2018). Highlighting the clinical impact of these comorbidities, studies conducted over the past 15 years in SLE patients from multiple regions around the world report that the presence of MetS is predictive of organ damage accrual as measured via the Systemic Lupus International Collaborating Clinics American College of Rheumatology Damage Index (SLICC/ACR DI) (Mok et al., 2018; Apostolopoulos et al., 2020; Ugarte-Gil et al., 2022). Together, these studies speak to a need for further investigation of how cardio-metabolic risk factors affect outcomes in SLE and whether SLE itself contributes to the development of MetS.

Animal models of SLE provide an opportunity to further study the interactions between SLE and MetS. Of the spontaneous mouse models of SLE, the female (New Zealand Black x New Zealand White) F1 mouse (NZBWF1) has been the most extensively characterized with respect to MetS phenotypes. Female NZBWF1 mice display hypertension, adiposity, hyperinsulinemia, and impaired glucose tolerance even on normal rodent chow (Ryan et al., 2006; Vilà et al., 2012). Feeding NZBWF1 mice a 45% kcal high-fat diet for 14 weeks accelerates the onset of albuminuria and worsens endothelial dysfunction (Gilbert and Ryan, 2011). A long-term high-fat diet has also been reported to induce development of atherosclerotic lesions in NZBWF1 mice (Hahn et al., 2010). Vasculitis, arterial lipid deposition and myocardial infarctions were also seen following feeding of an atherogenic diet to another spontaneous mouse model of lupus, the MRL/lpr mouse (Qiao et al., 1993). As such, these spontaneous mouse models of SLE appear vulnerable to the development of MetS and diet-induced cardiovascular complications, highlighting the translational relevance of the models.

Inducible models of SLE offer the opportunity to more easily probe the roles of specific genes by superimposing SLE onto a transgenic mouse of interest. A key pathway leveraged in multiple currently available inducible mouse models of SLE is toll-like receptor 7 (TLR7) activation. Enhanced signaling through toll-like receptors, including TLR7, has been implicated in promoting autoimmune disease (Farrugia and Baron, 2017). TLR7 is an X chromosome-encoded endosomal receptor that recognizes single-stranded RNA. Genome-wide association studies report polymorphisms in the TLR7 locus segregating with SLE (Shen et al., 2010; dos Santos et al., 2012) and more recently, a gain-of-function mutation in TLR7 was identified in a pediatric SLE patient (Brown et al., 2022). Proposed mechanisms by which TLR7 activation may contribute to SLE include through stimulating type I interferon production, expansion and plasma cell differentiation of autoreactive double-negative B cells, germinal center formation, and enhancing production of autoantibodies to RNA-containing antigens (Christensen et al., 2006; Subramanian et al., 2006; Lee et al., 2008; Jenks et al., 2018). As recently reviewed, an imbalance of the pathogenic role of TLR7 and protective role of TLR9 on effector function of B cells may also contribute to SLE (Fillatreau et al., 2021). The hydrocarbon oil pristane has been used to induce SLE in mice, working via a TLR7-dependent pathway (Lee et al., 2008). Whether the inducible pristane model of lupus is associated with features of MetS other than hypertension does not appear to have been explored. A more recently-developed inducible model of SLE is the treatment of mice with a TLR7 agonist such as imiquimod (Yokogawa et al., 2014). Taken together with the need to better understand the interaction between SLE and MetS, the purpose of the current study was to determine whether combined TLR7 agonist treatment and exposure to a high fat, high sucrose “Western diet” intervention affects the early-stage development of SLE or MetS in comparison to a high sucrose control diet.

## Materials and Methods

### Animal Procedures

All procedures were approved in advance by the University of Nebraska Medical Center Institutional Animal Care and Use Committee and were in agreement with the National Institutes of Health’s Guide for the Care and Use of Laboratory Animals. Female C57BL/6 mice were purchased from Jackson Laboratories (Bar Harbor, ME, Cat# JAX:000,664; RRID:IMSR_JAX:000,664), and with *n* = 8 group were housed four per cage on a 12-h light/dark cycle. Commencing at 12 weeks of age, mice were fed either a high-fat “Western” diet (HFD; fat 42% kcal, sucrose 34% kcal; Cat# TD88137, Teklad, United States) or control diet (fat 12.6% kcal, sucrose 34% kcal; Cat# TD05230, Teklad, United States) for 6 weeks. At the same time, mice were untreated or treated epicutaneously on the ear 3 times weekly with the TLR7 agonist imiquimod (IMQ) at 1.25 mg of 5% IMQ cream (Aldara, Valeant Pharmaceuticals, NJ, United States) following a method previously described by Yokogawa and others (Yokogawa et al., 2014), giving a total of 4 experimental groups.

Mice were placed into metabolic cages (Lab Products, Inc, Seaford, DE) weekly for 24 h to allow for timed measurement of food and water intake and urine output. On the day of euthanasia for tissue collection, mice were fasted for 5–6 h (8 a.m.–1 p.m.), and conscious fasting blood glucose level was measured by glucometer (ACCU-CHEK^®^, Aviva plus) in blood collected by pricking the tail with a sterile needle. Mice then were euthanized by overdose of isoflurane (VetOne, Boise, IND) and cervical dislocation, allowing terminal blood collection by cardiac puncture into a heparinized syringe. Spleen, kidney, gonadal fat, and blood were harvested for biochemical and histological analysis, with the left ventricle plus septum dissected and weighed, and tibia length was also measured.

### Plasma and Tissue Analysis

Blood samples were centrifuged at 10,000 g for 5 min at 4°C, and the plasma was collected and frozen at -80°C until analysis. Commercially available ELISA kits were used to determine the following in plasma according to the manufacturers’ instructions: insulin (Cat# EMINS, Invitrogen, Fredrick, MD, United States; per the manufacturer, the intra-assay coefficient of variation (CV) was <10%, inter-assay CV values were <12%, and detection range lower limit of 6.25 µIU/ml); leptin (Cat# KMC2281, Invitrogen; per the manufacturer, intra-assay CV was <8.48%, inter-assay CV was <9.89%, detection range lower limit of 93.8 pg/ml); total anti-nuclear antibodies (ANA) (Cat# 5210, Alpha Diagnostics International Inc, San Antonio, TX, United States); anti-dsDNA IgG concentrations (Cat# 5120, Alpha Diagnostics International Inc, San Antonio, TX, United States). Both autoantibody kits have a detection range lower limit of 50 U/ml. Urine albumin concentration was measured by ELISA (Albuwell M, Cat# 1011, Ethos Biosciences Inc, Logan Township NJ, United States; detection range lower limit of 0.156 µg/ml). Measurements for ELISAs were reported as concentrations determined via use of a standard curve except for ANA, where the need to dilute samples necessitated the calculation of a Positive Index instead. Samples were considered positive if the sample delta optical density (450–630 nm) was greater than a Positive Index, which was calculated as the mean delta optical density (450–630 nm) + 2 standard deviations of the untreated control diet group. For the purposes of statistical analysis of ANA levels, an Antibody Activity value was calculated for each sample by dividing the sample delta optical density (450–630 nm) by the Positive Index. Plasma high-density lipoprotein (HDL), low-density lipoprotein (LDL)/very low-density lipoprotein (VLDL), and total cholesterol were determined by colorimetric assay (Cat# ab65390, Abcam, Cambridge, MA, United States; detection range lower limit of 20 µg/ml).

### Statistical Analysis

All data are expressed as mean ± standard error of the mean (SEM). Statistical analysis was performed using GraphPad Prism v. 9.1.0 (RRID:SCR_002,798) using a two-way analysis of variance (ANOVA) testing for the main effects of dietary intervention (*P*_Diet_) or IMQ treatment status (*P*_IMQ_), and the interaction between diet and IMQ treatment (*P*_Diet*IMQ_). Where a significant interaction was observed, pairwise comparisons were made via Bonferroni post-hoc test, correcting for multiple hypothesis testing; *p* < 0.05 was considered statistically significant.

## Results

### Effect of Diet and Imiquimod Treatment on Body Morphology

**FIGURE 1:**
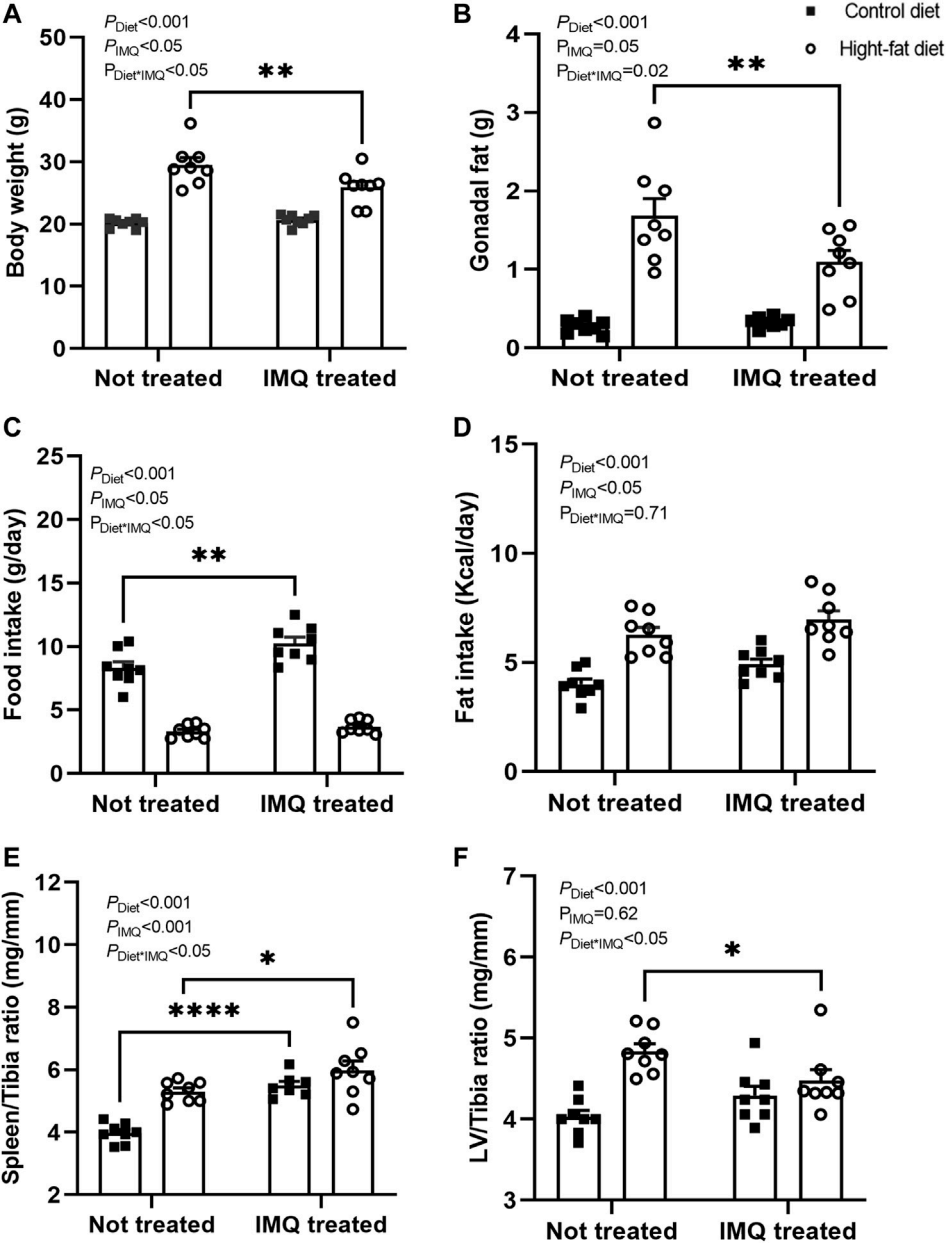
High-fat diet and imiquimod treatment affected body morphology. **(A)** Body weight and **(B)** gonadal fat pad mass were higher in high-fat diet mice compared to control diet mice after 6 weeks of treatment, with this effect mildly but significantly attenuated by imiquimod (IMQ) (**p < 0.001 by Bonferroni post-hoc comparison). **(C)** Average food intake calculated from weekly 24 h metabolic cage measurements was higher in control than high-fat diet mice, with a further small but significant increase in IMQ-treated versus untreated control diet mice. **(D)** Calculated average Kcal of fat intake was higher in high fat than control diet mice and significantly higher in IMQ than in untreated mice. **(E)** Spleen-to-tibia ratio was greater in both IMQ-treated and high-fat diet mice. **(F)** Left ventricle (LV)-to-tibia ratio was increased by fat diet with the effect mildly attenuated by IMQ (*p < 0.05 by Bonferroni post-hoc comparison). n = 8 per group with individual data points and mean ± SEM also shown. Data were compared by two-way ANOVA, with *P*_*D*iet_ = main effect of diet; *P*_IMQ_ = main effect of treatment; *P*_Diet*IMQ_ = interaction between diet and treatment. Bonferroni post-hoc comparisons: *p < 0.05, **p < 0.001, ****p < 0.001).

At the end of the 6-weeks treatment protocol, there was a significant difference in body weight between untreated and IMQ-treated mice overall (*P*_IMQ_ <0.05; Figure 1A). As expected, HFD-fed mice had significantly higher body weights compared with the control diet-fed mice (*P*_Diet_ < 0.001). IMQ treatment affected this response to diet (*P*_Diet*IMQ_ < 0.05), with HFD-fed IMQ-treated mice weighing significantly less than their untreated but HFD-fed counterparts (*p* < 0.05). Gonadal fat pad mass showed a similar pattern (Figure 1B), with fat accumulation observed in HFD-fed mice compared to control diet-fed mice (*P*_Diet_ < 0.001), and a statistically significant attenuation of fat deposition in the HFD-fed IMQ-treated mice (*p* < 0.05). Food intake was measured in individual mice for 24 h weekly, and the average of these 6 measurements is presented in Figure 1C. There was a significant difference in food intake between untreated and IMQ-treated mice overall (*P*_IMQ_ <0.05). Control diet-fed mice showed significantly greater food intake compared to HFD-fed mice (*P*_Diet_ < 0.001; Figure 1C), and this effect was slightly but significantly enhanced in IMQ-treated mice (*p* < 0.05). IMQ-treated mice overall showed significantly greater fat intake compared to untreated mice (*P*_IMQ_ < 0.05; Figure 1D). Calculated fat intake was significantly greater in HFD-fed mice than control diet-fed mice irrespective of treatment group (*P*_Diet_ < 0.05; Figure 1D). As an early marker of immune system activation, spleen-to-tibia ratio (Figure 1E) was significantly increased in IMQ-treated compared to untreated mice (P_IMQ_ < 0.001). HFD-feeding also increased spleen-to-tibia ratio (*P*_Diet_ < 0.001). Similarly, raw spleen weight was significantly higher in IMQ-treated mice irrespective of diet (*P*_IMQ_<0.001), and increased in HFD-fed mice irrespective of treatment group (*P*_Diet_ < 0.001), with splenomegaly similar in the two IMQ and untreated HFD groups (control diet untreated mice, 69 ± 2 mg; IMQ-treated control diet mice, 96 ± 3 mg; untreated HFD mice, 93 ± 2 mg; IMQ-treated HFD mice, 102 ± 4 mg). IMQ treatment *per se* did not significantly affect the left ventricle weight-to-tibia length ratio (*P*_IMQ_ = 0.62). HFD-fed animals overall showed increased left ventricle weight-to-tibia length ratio (*P*_Diet_ < 0.05; Figure 1F), an effect that was attenuated in IMQ-treated compared to untreated mice (*p* < 0.05).

### Effect of Diet and Imiquimod Treatment on Plasma ANA and dsDNA IgG Antibodies

For mice with sufficient plasma available, ANA positivity was observed in 0/6 control diet untreated mice, 3/7 control diet IMQ-treated mice, 1/6 untreated HFD mice, and 5/7 HFD IMQ-treated mice. All positive values were less than double the Positive Index (calculated Antibody Activity range of 1.00–1.88). Two-way ANOVA of these data revealed a significant overall effect of IMQ treatment to provoke ANA positivity (*P*_IMQ_ < 0.05), but no significant effect of diet (*P*_Diet_ = 0.7) or interaction between diet and treatment (*P*_Diet*IMQ_ = 0.8). There was no significant difference between groups in plasma dsDNA IgG concentrations, which were 3041 ± 647 U/ml in control diet untreated, 2755 ± 272 U/ml in control diet IMQ-treated, 2724 ± 286 U/ml in HFD untreated and 2607 ± 220 U/ml in HFD IMQ-treated (n = 4-8 due to insufficient plasma remaining for some individuals).

### Effect of Diet and Imiquimod Treatment on Albumin Excretion

Urinary albumin excretion at 6 weeks averaged 16 ± 4 µg/d in control diet untreated, 13 ± 2 µg/d in control diet IMQ-treated, 6 ± 1 µg/d in HFD untreated, and 4 ± 1 µg/d in HFD IMQ-treated mice. There was no significant effect of IMQ on albumin excretion (*P*_IMQ_ = 0.3), nor was there any significant interaction of diet and IMQ-treatment (*P*_Diet*IMQ_ = 0.7). Albumin excretion was, however, significantly lower in HFD-fed mice than in control diet mice (*P*_Diet_ < 0.05).

### Effect of Diet and Imiquimod Treatment on Fasting Glycemia

**FIGURE 2:**
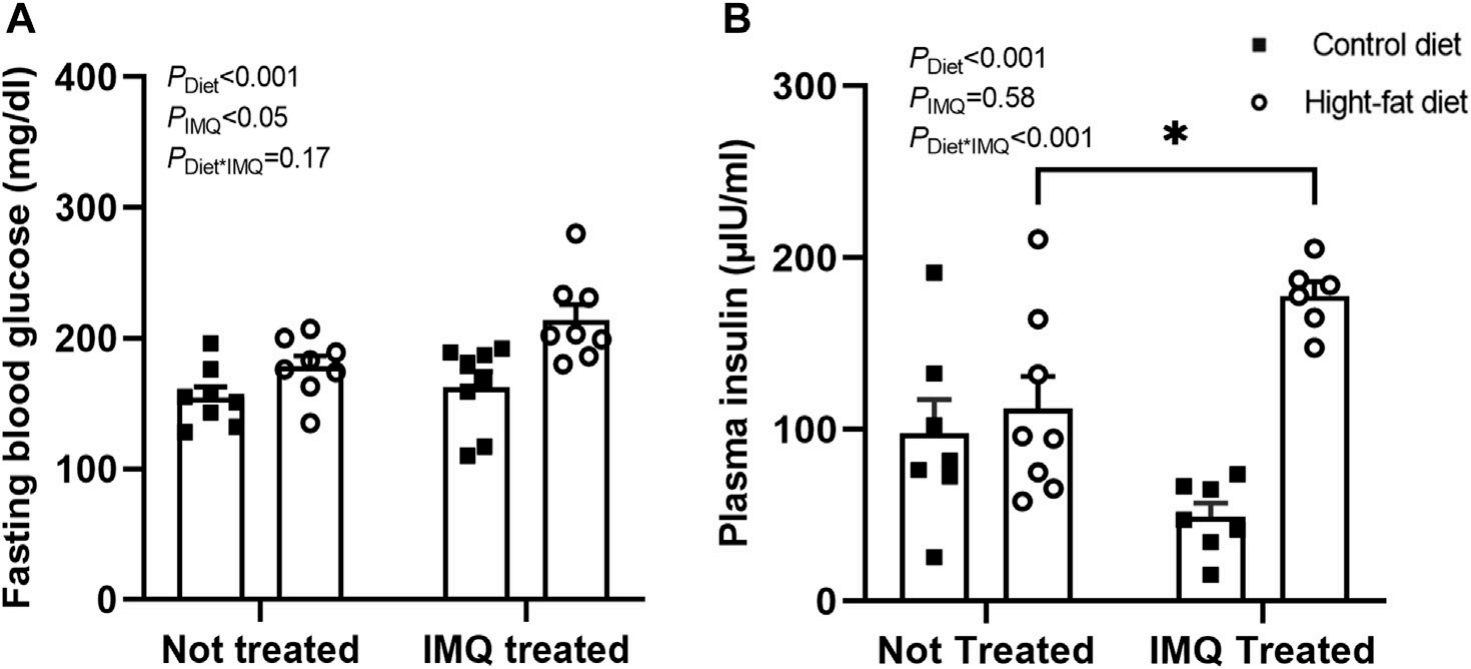
Imiquimod impairs glucose homeostasis. **(A)** Conscious fasting blood glucose after 6 weeks of treatment was significantly elevated in high-fat diet and IMQ-treated groups. **(B)** IMQ affected fasted plasma insulin concentration in a diet-dependent manner, with hyperinsulinemia observed only in IMQ-treated mice on a fat diet (*p < 0.05 by Bonferroni post-hoc test). Statistical comparisons are indicated in Figure 1. Data are shown for n = 6-8 per group due to insufficient plasma available for some mice.

After 6 weeks of treatment, both IMQ and HFD independently increased fasting blood glucose (*P*_IMQ_ < 0.05, *P*_Diet_ < 0.001; Figure 2A). However, there was no synergistic effect of these two stimuli (*P*_Diet*IMQ_ = 0.17). IMQ treatment had a dichotomous effect on plasma insulin concentration such that there was no significant main effect of IMQ when treatment groups were considered together (*P*_IMQ_ = 0.58; Figure 2B). Rather, IMQ-treatment affected plasma insulin concentration in a diet-dependent manner (*P*_Diet*IMQ_ < 0.001), with HFD-fed IMQ-treated mice displaying significant hyperinsulinemia relative to untreated HFD-fed mice (*p* < 0.05 by post-hoc comparison). Overall, HFD-fed mice showed significantly higher plasma insulin concentrations compared to control diet-fed mice (*P*_Diet_ < 0.001).

### Effect of Diet and Imiquimod Treatment on Plasma Leptin and Lipid-Related Parameters

**FIGURE 3:**
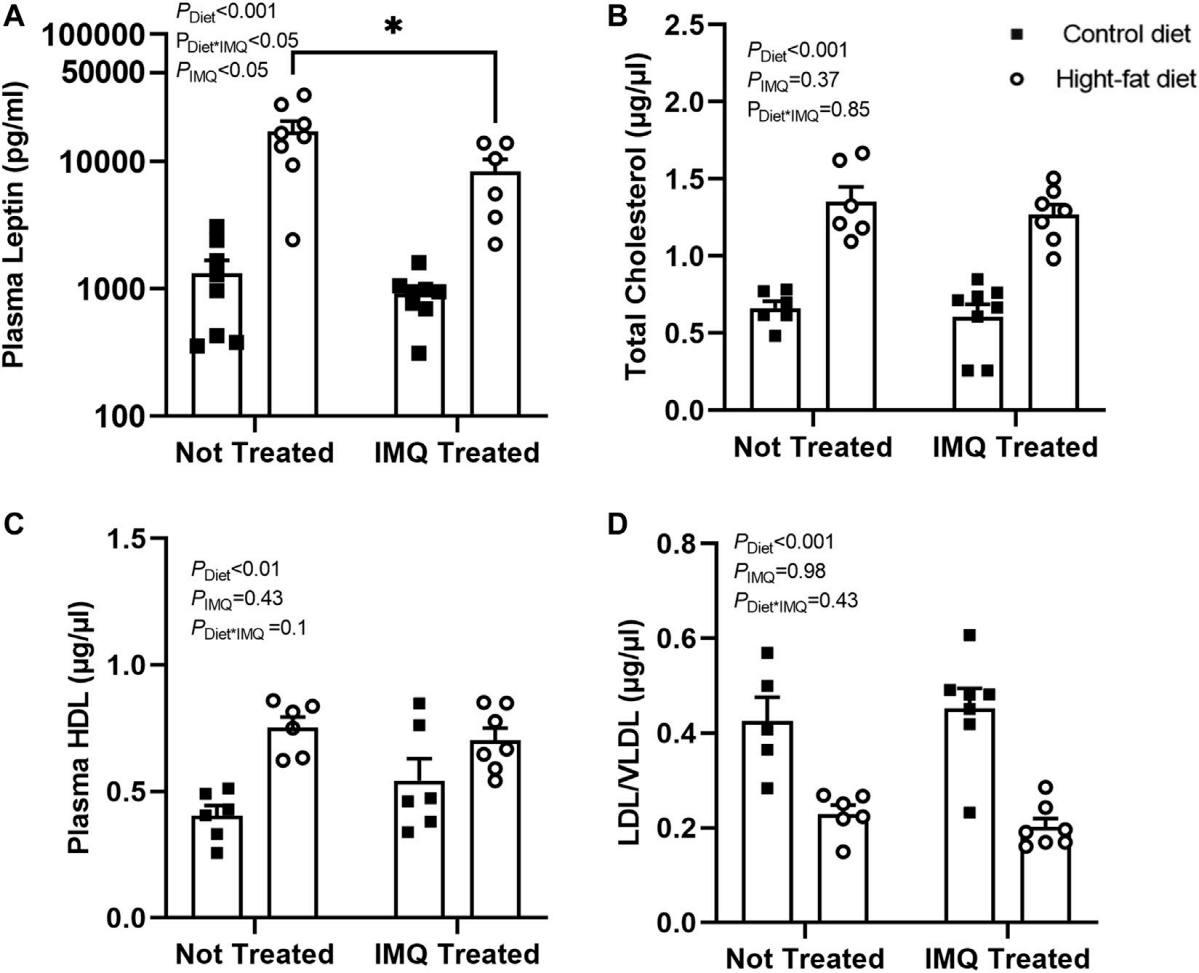
Imiquimod attenuates plasma leptin increases but does not affect diet-induced changes in cholesterol levels. **(A)** High-fat diet increased plasma leptin, although this effect was attenuated by IMQ (*p < 0.05). **(B)** Total cholesterol and **(C)** HDL cholesterol was increased by high-fat diet with no significant effect of IMQ. **(D)** High-fat diet mice had lower LDL/VLDL compared to control diet mice, but there was no effect of IMQ. Statistical comparisons are indicated in Figure 1. Data are shown for n = 6-8 per group due to insufficient plasma available for some mice.

Plasma leptin levels overall were lower in IMQ-treated compared to untreated mice (*P*_IMQ_ < 0.05), but the effect was diet-dependent (*P*_Diet*IMQ_ < 0.05; Figure 3A). As expected, plasma concentrations of leptin were significantly increased in the HFD-fed mice compared to the control diet-fed mice (*P*_Diet_ < 0.001). Similar to the effects seen on body weight and gonadal fat pad mass, the elevation of plasma leptin was attenuated slightly but significantly in the IMQ-treated HFD-fed mice compared to untreated HFD-fed mice (*p* < 0.05) but not significantly different between control diet-fed untreated and IMQ-treated mice. IMQ treatment did not affect total cholesterol (*P*_IMQ_ = 0.37; Figure 3B). HFD-fed mice had significantly higher total cholesterol levels in the plasma compared to control diet-fed mice (*P*_Diet_ < 0.001). IMQ did not significantly affect plasma HDL levels (*P*_IMQ_ = 0.43; Figure 3C). Plasma HDL was unexpectedly higher in HFD-fed mice compared to control diet-fed mice (*P*_Diet_ < 0.01). IMQ did not affect LDL/VLDL (*P*_IMQ_ = 0.98; Figure 3D), but this was higher among control diet mice compared to HFD-fed mice (*P*_Diet_ < 0.001).

## Discussion

Given the increased prevalence of MetS in SLE patients (Sun et al., 2017; Hallajzadeh et al., 2018) and the importance of MetS in promoting cardiovascular and renal disease, a better understanding of how these comorbidities interact is essential to improving the health of SLE patients. The goal of the present study was to test the early-stage effects of dual exposure of C57BL/6 mice to stimuli that promote the development of SLE and MetS. Based on the observation of elevated fasting blood glucose, hyperinsulinemia, adiposity, and development of splenomegaly, our data suggest that HFD plus IMQ treatment may provide such a model for further studying the synergistic interplay of the mechanisms underlying both conditions. Moreover, a striking finding of the current study was the apparent acceleration of the development of hyperinsulinemia in IMQ-treated HFD-fed mice. Previous studies of NZBWF1 mice revealed impaired glycemic control, hyperinsulinemia, adiposity, and hypertension even on normal rodent chow (Ryan et al., 2006; Vilà et al., 2012). Together, these findings suggest that factors associated with SLE or autoimmunity rather than iatrogenic effects of treatments may render patients more susceptible to developing MetS.

Whether TLR7 plays a role in MetS has not been extensively investigated, but a diffuse body of literature so far provides evidence that it might. TLR7 was among several differentially-upregulated genes identified in the livers of a nonalcoholic steatohepatitis model in minipigs that was induced by chronic feeding of a high-fat, high-sucrose diet (Xia et al., 2014). In a community-based observational study of the Framingham Heart Study Offspring Cohort, TLR7 mRNA levels in platelets isolated from women showed a small but statistically significant association with body mass index, total cholesterol to HDL ratio, and serum C-reactive protein (Koupenova et al., 2015). Conversely, male CD-1 mice fed a high-fat diet showed downregulation of TLR7 mRNA in liver and adipose tissue (Betanzos-Cabrera et al., 2012). TLR8 knockout mice have high endogenous TLR7 expression and activity that promotes a lupus-like phenotype (Demaria et al., 2010). The impaired glucose tolerance and hepatic inflammation that developed in TLR8 knockout mice after feeding a 60% kcal fat diet for 18 weeks was attenuated by double knockout of both TLR7 and 8 (Hanna Kazazian et al., 2019). TLR7 knockout mice also had improved glucose tolerance compared to wild-type control mice (Hanna Kazazian et al., 2019). As described above, NZBWF1 mice display hyperinsulinemia and impaired glucose tolerance (Ryan et al., 2006). The autoimmunity and glomerulonephritis observed in NZBWF1 mice are partially TLR7-mediated (Murakami et al., 2021), but whether the hyperinsulinemia and impaired glucose tolerance are also related to TLR7 activation has not been studied to our knowledge. Together with our data, these studies show potential for the involvement of TLR7 signaling in the development of dysglycemia and hyperinsulinemia, although the mechanisms underlying these effects remain to be further elucidated. A role for TLR7 in promoting inflammation of the liver (Hanna Kazazian et al., 2019) may be relevant to the disturbances of glycemic control in MetS and the high prevalence of dysglycemia and hyperinsulinemia SLE, given the liver’s central role in whole-body glucose homeostasis; further investigation in this area appears warranted.

Excessive body weight and adiposity are key components of the MetS and associated with the future development of insulin resistance (Hayashi et al., 2008; He Y. et al., 2020). Further, obesity appears to worsen autoimmune disorders in a number of incompletely understood ways, including promoting autoantibody production and secretion of adipokines such as leptin that promote inflammation (Tsigalou et al., 2020). The status of leptin in SLE has been somewhat controversial, but a 2020 meta-analysis (Yuan et al., 2020) reported overall elevated plasma leptin levels among female SLE patients, and some indication that a leptin receptor gene variant (rs1137101) may confer increased susceptibility to SLE. An increased frequency of leptin receptor-positive B cells in SLE patients and an effect of leptin to promote B cell cytokine production and differentiation to highly IgG and IgM-secreting plasma cells was also recently reported (Chen et al., 2022 Clin Exp Rheum). In the current study, the body weights and visceral (gonadal) fat deposition were found to be elevated in HFD-fed animals when compared with control animals, but IMQ-treatment mildly attenuated this difference. Similarly, plasma leptin concentrations were also increased by HFD but mildly attenuated within the same group. This apparent mild inhibitory effect of TLR7 activation on development of diet-induced obesity and hyperleptinemia contrasts with a mild protective effect of TLR7 knockout noted by Hanna Kazadian et al. (Hanna Kazazian et al., 2019). The reasons for this difference remain currently unclear, however the slightly but significantly reduced adiposity in IMQ-treated HFD mice does not seem to have been caused by a reduction of food intake. Nonetheless, HFD-feeding provides an environment of increased adiposity and hyperleptinemia, allowing further study of interactions between obesity and TLR7-mediated autoimmunity.

SLE patients have altered lipid profiles characterized by increased triglycerides and reduced HDL cholesterol concentrations (Chung et al., 2007). Although triglycerides were not measured in the current study due to insufficient plasma for analysis, we found increased total cholesterol levels among HFD-fed mice compared with control diet-fed mice. Interestingly, HDL rather than LDL/VLDL levels were elevated in the HFD-fed mice. Per the manufacturer’s specification sheet, the main fat source in the Teklad TD88137 HFD is anhydrous milk fat. An elevation of HDL cholesterol in C57BL/6 mice fed a high fat diet where the fat came predominantly from milk fat has been reported previously by others (Podrini et al., 2013). NZBWF1 mice exposed to a high fat “Western” diet also developed elevated HDL cholesterol, which included so-called “pro-inflammatory” HDL cholesterol that has pro-oxidant rather than antioxidant properties, as described by (Hahn et al., 2010). Whether the elevated HDL cholesterol measured in the HFD groups in the current study also included pro-inflammatory HDL cholesterol is unknown, as we had insufficient plasma available to determine this. Regardless and in contrast to effects on plasma insulin, IMQ treatment did not significantly affect any of the cholesterol parameters measured in the current study. He et al. (He M. Q. et al., 2020) found that LDL cholesterol and total cholesterol were more elevated after 9 weeks of a low sucrose, 60% fat diet protocol in C57BL/6 mice than after 5 weeks; therefore, a longer duration of study and perhaps a different diet composition to that used in the current study would be beneficial for studying the impact of TLR7-driven autoimmunity on other components of dyslipidemia.

Various dietary approaches have been used to model MetS in rodents, with chow containing high fat and high carbohydrate (often fructose) used singly or in combination, or drinking water containing sucrose or fructose (Wong et al., 2016). The high-fat, high-sucrose “Western” diet used in the current study (TD88137) was chosen rather than high fat alone in part to incorporate excess dietary sugar intake, which is common in the MetS population at large. Highlighting the importance of incorporating both high fat and high sucrose in understanding inflammatory outcomes, a recent study of IMQ-induced psoriasiform dermatitis noted an exaggerated inflammatory response to a similar high-fat, high-sucrose western diet to the one used in the current study than to a diet higher in fat alone (Yu et al., 2019). A caveat to the interpretation of differences between our control and HFD groups is, however, that the control diet used in the current study was a modified version of the TD88137, containing a matched sucrose level. A paired, low sucrose diet was not available. Selection of this control diet was done to allow the best possible consistency in other dietary components such as protein sources (casein). As such, the diet-induced differences between the groups in the current study are presumptively high fat-driven.

Splenomegaly is a common feature of SLE models (Du et al., 2015) and observed in both the IMQ-treated mouse groups in the current study, as well as by Yokogawa et al. (Yokogawa et al., 2014). Anti-dsDNA autoantibodies are common in SLE and typical for lupus nephritis, although autoantibodies also develop against other nuclear components. Indeed, the suite of autoantibodies developed (and organ involvement) differs in a strain- and genetic background-dependent manner in both spontaneous models such as the NZBWF1 as well as in inducible pristane and IMQ models (Reeves et al., 2009; Yokogawa et al., 2014). Yokogawa et al. (Yokogawa et al., 2014) reported an increase in anti-dsDNA autoantibodies in FVB/N and BALB/c mice after 4 weeks of IMQ treatment; data for C57BL/6 mice were not reported. We did not detect a significant rise in anti-dsDNA autoantibodies in female C57BL/6 mice in the present 6-weeks study. Similar to our study, treatment of male and female C57BL/6 mice with another TLR7 agonist, resiquimod, over a similar time-frame promoted splenomegaly, no significant rise in dsDNA autoantibodies but a significant increase in ANA (Wirth et al., 2020). Similarly to the absence of albuminuria in the C57BL/6 mice treated with IMQ in the current study, albuminuria had not developed after 5 weeks of resiquimod treatment of C57BL/6 mice (Wirth et al., 2020). Wirth and others (Wirth et al., 2020) did, however, note early mortality with 67% loss of female C57BL/6 mice by 6 weeks, but this is not something that we encountered in the current study or with longer treatment of transgenic mice on a C57BL/6 background in other ongoing work in our laboratory (unpublished data, not shown). The IMQ model is capable of producing glomerular Ig deposition and glomerulonephritis in other genetic backgrounds such as BALB/c and FVB/N (Yokogawa et al., 2014). Together, these data are consistent with TLR7 agonists promoting features of SLE in a strain-dependent manner, with additional variability within strains but between institutions possible for reasons that are as yet unclear.

Hypertension is an important risk factor for cardiovascular disease and progression of renal damage, and is prevalent in SLE patients. Female BALB/c mice treated with IMQ display elevated blood pressure as measured by tail-cuff plethysmography after 6 weeks (Robles-Vera et al., 2020). We did not measure arterial blood pressure in the present study and so cannot draw any conclusions regarding whether blood pressure was increased in any group. We did not, however, detect a significant effect of IMQ to induce left ventricular hypertrophy. The mild attenuation of the HFD-induced left ventricular hypertrophy in the IMQ-treated group is consistent with the overall attenuation of HFD effects on body weight and fat pad mass. The susceptibility of C57BL/6 mice to induction of hypertension by IMQ treatment remains to be determined.

The present study found that co-treatment of female C57BL/6 mice with HFD and IMQ induced dysglycemia, hyperinsulinemia, adiposity, and splenomegaly and ANA, indicating the induction of MetS and early stages of autoimmunity. This model may be useful for studying the role of TLR7 signaling in promoting or accelerating the development of dysglycemia and hyperinsulinemia in the context of diet-induced MetS.

## Data Availability

The raw data supporting the conclusion of this article will be made available by the authors, without undue reservation.
